# Thermal dynamics and electronic temperature waves in layered correlated materials

**DOI:** 10.1038/s41467-021-27081-2

**Published:** 2021-11-25

**Authors:** Giacomo Mazza, Marco Gandolfi, Massimo Capone, Francesco Banfi, Claudio Giannetti

**Affiliations:** 1 Department of Quantum Matter Physics, University of Geneva, Quai Ernest-Ansermet 24, 1211 Geneva, Switzerland; 2 CNR-INO, Via Branze 45, 25123 Brescia, Italy; 3 Department of Information Engineering, University of Brescia, Via Branze 38, 25123 Brescia, Italy; 4 Scuola Internazionale Superiore di Studi Avanzati (SISSA) and CNR-IOM Democritos National Simulation Center, Via Bonomea 265, 34136 Trieste, Italy; 5 FemtoNanoOptics group, Université de Lyon, CNRS, Université Claude Bernard Lyon 1, Institut Lumière Matière, F-69622 Villeurbanne, France; 6 Dipartimento di Matematica e Fisica, Università Cattolica del Sacro Cuore, Via Musei 41, I-25121 Brescia, Italy; 7 Interdisciplinary Laboratories for Advanced Materials Physics (I-LAMP), Università Cattolica del Sacro Cuore, Via Musei 41, I-25121 Brescia, Italy

**Keywords:** Electronic properties and materials, Nanoscale materials

## Abstract

Understanding the mechanism of heat transfer in nanoscale devices remains one of the greatest intellectual challenges in the field of thermal dynamics, by far the most relevant under an applicative standpoint. When thermal dynamics is confined to the nanoscale, the characteristic timescales become ultrafast, engendering the failure of the common description of energy propagation and paving the way to unconventional phenomena such as wave-like temperature propagation. Here, we explore layered strongly correlated materials as a platform to identify and control unconventional electronic heat transfer phenomena. We demonstrate that these systems can be tailored to sustain a wide spectrum of electronic heat transport regimes, ranging from ballistic, to hydrodynamic all the way to diffusive. Within the hydrodynamic regime, wave-like temperature oscillations are predicted up to room temperature. The interaction strength can be exploited as a knob to control the dynamics of temperature waves as well as the onset of different thermal transport regimes.

## Abstract (web summary)

Quantum correlated materials offer a promising platform for the study of unconventional heat transport. Here the authors theoretically investigate heat transport in a layered correlated material, triggered by an ultrafast excitation, and predict various transport regimes controlled by correlations.

## Introduction

The capability to access ultrafast thermal dynamics recently gave access to striking phenomena that take place in materials at the nanoscale before complete local energy equilibration among heat carriers is achieved^1–5^. For instance, non-Fourier heat transport regimes have been reported for hot spots dimensions inferior to the phonon mean free-path^6–8^, in which energy is ballistically carried point to point, or have been engineered via nano-patterning of dielectric substrates^9–11^. As a consequence of the existence of two non-thermal populations, wave-like thermal transport, often referred to as second sound^12,13^, has been predicted in graphene, both in the frame of microscopic^14–17^ and macroscopic models^18^. Temperature wave-like phenomena have been recently observed at high temperatures in graphene^19^ and 2D materials^20^ on sub-nanosecond timescales and scheme for their coherent control have been proposed^21^. So far most of the effort has been devoted to phononic non-Fourier heat transport^17,19,22–26^, where, only recently, a theoretical framework, covering on equal footing Fourier diffusion, hydrodynamic propagation, and all regimes in between, has been proposed^27^. On the contrary, electronic non-Fourier heat transport remains relatively unexplored^18,20,28,29^.

In this work, we propose layered correlated materials (LCM) as the ideal platform to access the entire spectrum of unconventional electronic heat transport regimes. In quantum correlated materials, the strong electronic interactions give rise to emerging many-body properties, such as collective and decoupled diffusion of energy and charge^30,31^. Tuning the interaction strength thus opens the possibility to investigate transport regimes with no counterpart in conventional weakly-interacting materials^32,33^.

**Fig. 1:**
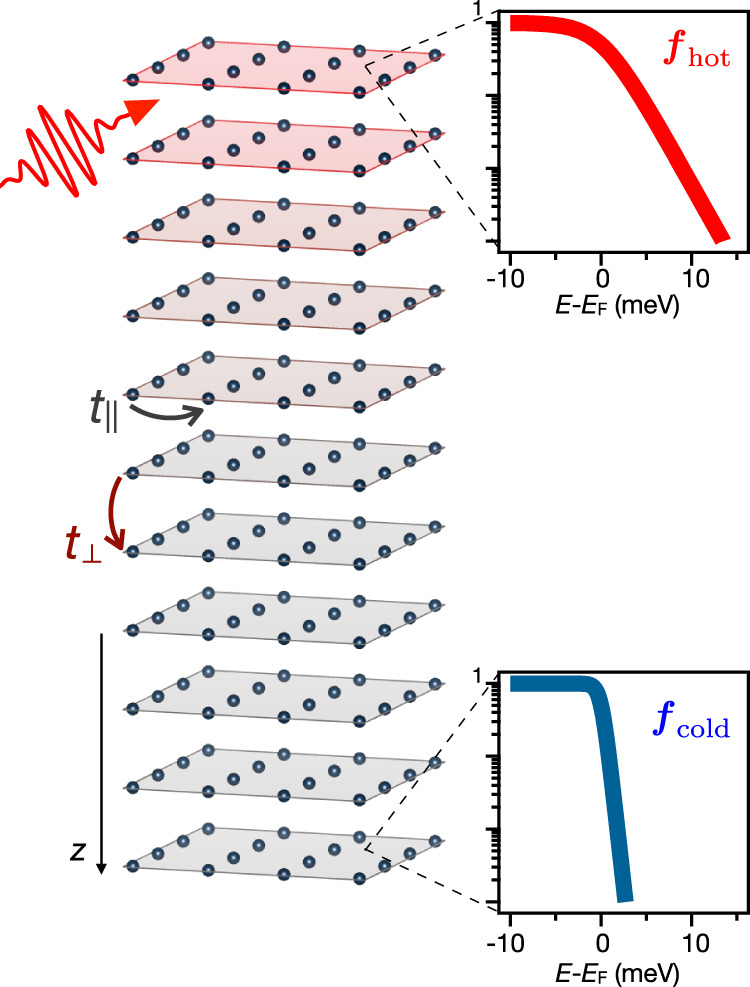
Setup. Cartoon of the layered correlated material impulsively excited on the top surface by ultrafast light pulses. We assume that the excitation drives a fast thermalization of the electronic population establishing an electronic temperature *T*_hot_ on the topmost layers of the sample. The right panels display the electronic distributions at different depths after the equilibration step (see text).

We consider an impulsive excitation on the surface of a LCM characterized by a strong local Coulomb interaction *U* (see Fig. 1). The interaction *U* can drive fast local thermalization processes leading to the rapid build up of a hot intra-layer electronic temperature before relaxation via slower scattering paths takes place. At the same time, the interaction leads to heavier quasiparticles with enhanced effective mass m^*^ and a reduced kinetic energy. As a consequence, energy propagation across the layers is expected to slow down for increasing *U*. Overall the interaction *U*, together with the anisotropy of the inter-layer and intra-layer hopping terms, may thus act as a tuning parameter to control the relative inter- and intra-layer energy exchange processes in LCM. Eventually, as the interaction increases, the two processes can effectively decouple, thus allowing for unconventional electronic heat transport regimes to occur on the ultrashort space and timescales.

We investigate the possibility for unconventional heat transport regimes by focusing on the impulsive thermal dynamics of the layered single-band Hubbard model, which represents a general framework for understanding the effects of electronic interactions in a large family of correlated materials. We show that on sub-picosecond timescales the electronic heat transfer is initially characterized by ballistic wavefront propagation, followed by an hydrodynamic regime, which eventually evolves into conventional Fourier heat transfer on longer timescales. In the hydrodynamic regime, we predict that LCM may sustain temperature wave oscillations at THz frequencies and up to ambient temperature.

In order to contextualize the present concepts within the frame of real systems and to connect with the realm of technologically relevant materials, we focus on the correlated metal SrVO_3_ (SVO). SVO is a paradigmatic representative of the wider class of correlated transition metal oxides (TMOs) and it has been proposed as a platform for a wealth of potential technological applications ranging from ideal electrode materials^34^, to Mott transistors^35^ and transparent conductors^36^. We argue that the degree of correlation of SVO, as measured by the interaction strength, is such that ballistic transport first, and wave-like thermal transport afterwords, are accessible on the sub-picosecond timescale. Our results, together with the possibility of heterostructuring TMO to atomic layer accuracy, promote these materials to ideal building blocks for nanothermal device architectures based on non-Fourier heat transport.

The present work rationalizes the microscopic interactions underlying unconventional electronic heat transfer phenomena in LCM. Our findings enlarge the functionalities of quantum materials^32,33^ to the realm of nanoscale heat transport^37^, beyond the case of radiative energy transfer^38–40^.

## Results and discussion

### Model and observables

The Hamiltonian of the layered Hubbard model reads:

1 $$H=\mathop{\sum }\limits_{n=1}^{L}{h}_{n}+\mathop{\sum }\limits_{n=1}^{L-1}{\tau }_{n,n+1}$$

with

2 $${h}_{n}=\mathop{\sum}\limits_{ < i,j > \sigma }{t}_{\parallel }{c}_{in\sigma }^{{{{\dagger}}} }{c}_{jn\sigma }^{}+U\mathop{\sum}\limits_{i}{n}_{in\uparrow }{n}_{in\downarrow }$$

and

3 $${\tau }_{n,n+1}=\mathop{\sum}\limits_{\sigma }{t}_{\perp }{c}_{in\sigma }^{{{{\dagger}}} }{c}_{in+1\sigma }^{}+h.c.$$

where ${c}_{in\sigma }^{{{{\dagger}}} }$ is a fermionic creation operator for an electron with spin *σ* at the site *i* belonging to the layer indexed by *n*, which ranges from 0 to *L*. *t*_∥_ and *t*_⊥_ represent, respectively, the intra- and inter-plane hopping amplitudes. The sum of the in-plane hopping term runs over pairs of nearest neighbouring sites and we introduce the number operator ${n}_{in\sigma }={c}_{in\sigma }^{{{{\dagger}}} }{c}_{in\sigma }^{}$. We assume in-plane translational invariance so that we can introduce an in-plane momentum **k** = (*k*_*x*_, *k*_*y*_) and recast ${h}_{n}={\sum }_{{{{{{{{\bf{k}}}}}}}}\sigma }\epsilon ({{{{{{{\bf{k}}}}}}}}){c}_{{{{{{{{\bf{k}}}}}}}}n\sigma }^{{{{\dagger}}} }{c}_{{{{{{{{\bf{k}}}}}}}}n\sigma }^{}+U{\sum }_{i}{n}_{in\uparrow }{n}_{in\downarrow }$ with $\epsilon ({{{{{{{\bf{k}}}}}}}})=-2{t}_{\parallel }(\cos ({k}_{x}a)+\cos ({k}_{y}a))$ and *a* the lattice spacing. We fix the chemical potential in order to have an average occupation of one electron per site (half-filling) corresponding to the perfect particle-hole symmetric case. As a consequence, the total number of electrons per layer is conserved during the dynamics. This choice allows us to model energy transport in the absence of mass and charge transport. We consider the model parameters *t*_⊥_ = *t*_∥_ = 60 meV and *U* = 0.65 eV, which correspond to an interaction-driven mass renormalization *m*/m^*^ ≃ 0.3, and a lattice spacing *a* = 5 Å. This value of the effective mass renormalization is consistent with experimental estimates for SrVO_3_.

We study the non-equilibrium thermal dynamics in the frame of model (1) by means of a time-dependent variational approach based on the generalized Gutzwiller approximation for layered systems^41,42^ (see Methods). This approach provides a versatile tool for describing in a non-perturbative way the dynamics in the Hubbard model, which is governed by the interplay between the hopping terms *t*_∥_, *t*_⊥_ and the local Coulomb interaction *U*. In this framework, the dynamics is described by using a variational density matrix $\hat{\rho }(t)$ with the structure^43,44^ $\hat{\rho }(t)\equiv {{{{{{{\mathcal{P}}}}}}}}(t){\hat{\rho }}_{* }(t){{{{{{{{\mathcal{P}}}}}}}}}^{{{{\dagger}}} }(t)$, where ${\hat{\rho }}_{* }$ is a density matrix which describes the dynamics of quasiparticles through an effective non-interacting Hamiltonian, and ${{{{{{{\mathcal{P}}}}}}}}={\prod }_{i}{{{{{{{{\mathcal{P}}}}}}}}}_{i}$ are projectors onto the local Hilbert space at site *i* which control the relative weights of the local many-body configurations. The mutual feedback between the dynamics of the localized degrees of freedom and the low-energy quasiparticle excitations results in an effective mass renormalization m^*^ = *m*/*Z*, which is controlled by the interaction *U* through the quasiparticle weight *Z*(*U*). In the non-interacting limit *Z*(*U* = 0) = 1, whereas at finite interaction *Z* < 1 and decreases as a function of *U*. Eventually, for a critical interaction strength, *U*_*c*_, the system undergoes a metal-to-insulator Mott transition, corresponding to a vanishing quasiparticle weight, i.e. *Z*(*U*_*c*_) → 0. In this regime, quasiparticle excitations are completely suppressed and the dynamics becomes dominated by high-energy incoherent excitations at energies ~ *U*^45^. In this work we focus on the thermal dynamics of hot quasiparticles in the correlated metal regime, where *Z* is finite but significantly smaller than one.

The thermal dynamics is triggered by a sudden increase of the electronic temperature localized within the first few surface layers of the LCM as can be achieved, for instance, by excitation with a femtosecond light pulse^28^. We mimic the impulsive excitation by considering the two-step non-equilibrium protocol (see Methods): first, we initialize the system in a non-equilibrium state characterized by two-electronic populations at two different temperatures. In the second step, we consider the unitary evolution of the initialized state, as regulated by the Hamiltonian (1). The initialization steps consists in a preliminary dynamics starting from the zero temperature state in which each layer *n* is independently coupled with baths of non-interacting electrons at temperatures *T*_*bath*_(*n*). All the layers are at a base temperature *T*_*bath*_(*n*) = *T*_*c*0_ except the five topmost which are set at *T*_*bath*_(*n*) = *T*_*h*_ ≫ *T*_*c*0_, for *n* = 1, …, 5, a temperature characterizing the hot electrons. In the notation *T*_*c*0_, the subscript “c” stands for cold and “0” indicates the instant right after the impulsive excitation, whereas the subscript “h” in *T*_*h*_ stands for hot. Throughout the paper, the temperatures used in the equilibration step are *T*_*c*0_ ≃ 35 K for the cold temperature in the bulk and *T*_*h*_ = 10 × *T*_*c*0_ for the hot temperature in the topmost layers.

The end of the equilibration step defines the instant of time *t* = 0. At *t* = 0, we initialize the density matrix with the density matrix obtained at the end of the equilibration step $\hat{\rho }(t=0)={\hat{\rho }}_{{{{{{{{\rm{equ}}}}}}}}}$. We switch off all the couplings to the baths and let the initialized density matrix unitary evolve for *t* > 0. We refer the reader to the *Methods* section for further details on the non-equilibrium protocol.

**Fig. 2:**
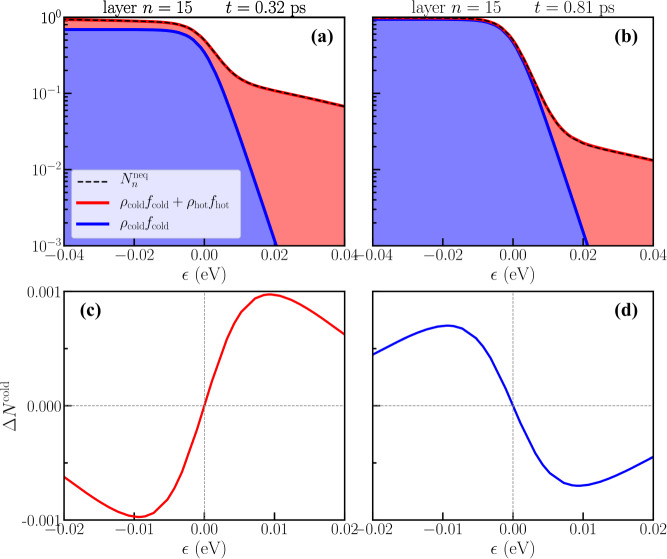
Non-equilibrium distribution functions decomposition. Non-equilibrium distribution functions, Eq. (4), decomposed in terms of the cold and hot Fermi-distribution functions. Data correspond to the non-equilibrium distribution functions for the layer *n* = 15 at two instants of time *t* = 0.32 ps (left panels) and *t* = 0.81 ps (right panels). Top panels **a, b**: *ρ*_cold_*f*_cold_ (blue lines) and sum of the contributions *ρ*_cold_*f*_cold_ + *ρ*_hot_*f*_hot_ (red line) compared with the non-equilibrium distribution functions ${N}_{n}^{{{{{{{{\rm{neq}}}}}}}}}$ (dashed black line), obtained by the numerical simulation. For simplicity we removed the layer and time index from the labels. The blue/red shaded regions helps to visualize the cold and hot contributions to the non-equilibrium distribution functions. Bottom panels **c, d**: Δ*N*_cold_, difference between the cold distribution function at the time *t*, and the initial one at temperature *T*_*c*0_.

At positive times, we study thermal transport by tracking the time evolution of the electronic temperature and of the heat flux at each layer. The electronic temperature is extracted from the time and layer-dependent quasiparticle non-equilibrium distribution functions ${N}_{n}^{{{{{{{{\rm{neq}}}}}}}}}(\epsilon ,t)$ (see Methods). For any instant of time *t* > 0, and for any layer index *n*, we find upon fitting that ${N}_{n}^{{{{{{{{\rm{neq}}}}}}}}}(\epsilon ,t)$ can be expressed as a superposition of two equilibrium Fermi distributions: (i) a hot distribution at the temperature *T*_*h*_, fixed by the initial equilibration temperature in the five topmost layers, and of weight *ρ*_hot_(*n*, *t*); (ii) a cold distribution characterized by a time- and layer-dependent temperature *T*(*n*, *t*) and of weight *ρ*_cold_(*n*, *t*) ≡ 1 − *ρ*_*hot*_(*n*, *t*)

4 $${N}_{n}^{{{{{{{{\rm{neq}}}}}}}}}(\epsilon ,t)={f}_{{{{{{{{\rm{hot}}}}}}}}}{\rho }_{{{{{{{{\rm{hot}}}}}}}}}(n,t)+{f}_{{{{{{{{\rm{cold}}}}}}}}}(n,t)\left[1-{\rho }_{{{{{{{{\rm{hot}}}}}}}}}(n,t)\right],$$

with 0 ≤ *ρ*_hot_(*n*, *t*) ≤ 1, *f*_hot_ = *f*(*ϵ*, *T*_*h*_) and *f*_cold_(*n*, *t*) = *f*(*ϵ*, *T*(*n*, *t*)). For any instant of time *t* and layer index *n*, we fit *N*^neq^(*ϵ*, *t*), computed via Eq. (19), with the expression given by Eq. (4), *ρ*_hot_(*n*, *t*) and *T*(*n*, *t*) being the only two fitting parameters. While the temperature of the hot electrons is fixed at *T*_*h*_, the temperature of the remaining 1 − *ρ*_hot_(*n*, *t*) fraction of electrons in the “cold” state *T*(*n*, *t*) changes in time. In Fig. 2 we show the decomposition, Eq. (4), for the layer *n* = 15 and two instants of time at *t* = 0.32 ps and *t* = 0.81 ps for which the non-equilibrium distribution functions are described by different populations of hot electrons, and different cold temperatures *T*(*n*, *t*).

Remarkably, the effective electronic temperatures at *t* = 0.32 ps and *t* = 0.81 ps extracted from the fit of Eq. (4) to ${N}_{n}^{neq}(\epsilon ,t)$ result to be, respectively, larger and smaller than the initial *T*_*c*0_. In order to demonstrate that these oscillations of *T*(*n*, *t*) are not an artifact related to the fitting procedure, we report in Fig. 2c, d direct evidence of temperature oscillations in the calculated distribution function. More specifically, we subtracted the contribution of the hot electrons (*ρ*_*hot*_(*n*, *t*)*f*_*hot*_) from the total distribution function ${N}_{n}^{neq}(\epsilon ,t)$, as calculated from the unitary dynamics. The normalized cold distribution can be thus expressed as ${N}_{n}^{{{{{{\mathrm{cold}}}}}}}(\epsilon ,t)=[{N}_{n}^{neq}(\epsilon ,t)-{\rho }_{{{{{\mathrm{{hot}}}}}}}(n,t){f}_{{{{{{\mathrm{hot}}}}}}}]/[1-{\rho }_{{{{{{\mathrm{hot}}}}}}}(n,t)]$. The difference between ${N}_{n}^{{{{{{\mathrm{cold}}}}}}}(\epsilon ,t)$ and the initial Fermi distribution, i.e. ${{\Delta }}{N}_{n}^{{{{{{\mathrm{cold}}}}}}}={N}_{n}^{{{{{{\mathrm{cold}}}}}}}(\epsilon ,t)-f(\epsilon ,{T}_{c0})$, clearly shows a change of occupation in the vicinity of the Fermi level which resembles a broadening (narrowing) of a Fermi distribution at *t* = 0.32 (0.81) ps. These data directly demonstrate that, during the dynamics, the electronic distribution changes in a complex oscillatory way, which can be accounted for by the two-temperature effective model described by Eq. (4). This fact allows for a clear physical interpretation of the transient propagation of energy and a practical definition of a local time-dependent electronic temperature. Initially, the perturbation creates a population of hot electrons described by *ρ*_hot_(*n*, *t*), which propagates across the layers. The interaction between the cold electrons on each layer and the hot electrons propagating through the system creates a modulation of the temperature of the cold electronic population, encoded in the spatio-temporal evolution of *T*(*n*, *t*). In the rest of the manuscript, we will refer to this quantity as the cold electronic temperature, bearing in mind that *T*(*n*, *t* = 0) = *T*_*n*_, with *T*_*n*_ the bath temperatures defined in the initialization step.

We extract the heat flux by considering the time evolution of the energy density defined for each layer as

5 $${E}_{n}(t)\equiv {{{{{{{\rm{Tr}}}}}}}}[\hat{\rho }(t)({h}_{n}+{\tau }_{n,n+1})]$$

where $\hat{\rho }(t)$ represents the time-evolved Gutzwiller density matrix. At positive times the density matrix evolves under unitary dynamics, so that energy is conserved. As a consequence, there exists a continuity equation that relates the time evolution of the local energy density *E*_*n*_ to the divergence of the current associated with the transport of energy, namely the heat flux. Therefore, by starting from the dynamics of the energy density, we compute the heat flux *q*_*n*_ at layer *n*, and along the *z*-direction perpendicular to the planes, by applying the continuity equation

6 $$\frac{\partial {q}_{n}}{\partial z}+\frac{\partial {E}_{n}}{\partial t}=0$$

where the discrete spatial derivative is defined with respect to the inter-layer distance *a*, $\frac{\partial {q}_{n}}{\partial z}=({q}_{n+1}-{q}_{n})/a$.

### Ultrafast thermal dynamics

**Fig. 3:**
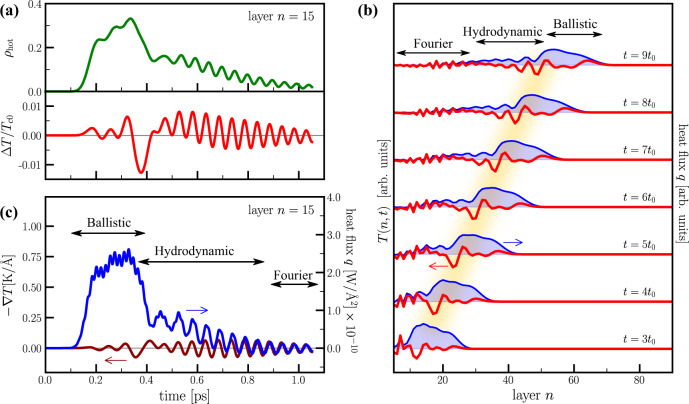
Sub-picosecond thermal dynamics. **a** Dynamics of the hot-electron population (top) and relative temperature variation of the “cold” electronic population (bottom) recorded on layer *n* = 15. **b** Layer profiles of the temperature of the “cold” electronic population (red, left axis) and of the heat flux (blue, right axis) at different instant of times. The blurred yellow band highlights the wave packet of temperature oscillations that follows the ballistic front. **c** Dynamics of the heat flux (blue, right axis) and temperature gradient of the “cold” electronic population (brown, left axis) on the layer *n* = 15. Arrows indicates the three regimes of thermal transport discussed in the main text. Panels **a** and **c** share the same *x*-axis. For simplicity the labels are shown only in the *x*-axis of panel **c**.

We now show how this model offers the possibility to access different regimes of non-conventional heat transport on the sub-picosecond timescale. Each regime will be then separately discussed in the following. Figure 3a reports the results for the time evolution of the hot population weight and the local electronic relative effective temperature variation, Δ*T*/*T*_*c*0_(*n*, *t*) with Δ*T* = *T*(*n*, *t*) − *T*_*c*0_, recorded on layer *n* = 15, which we take as representative of the inner region of the slab. For times 0 < *t* ≲ 150 fs, both *ρ*_hot_(15, *t*) and *T*(15, *t*) remain fixed to the equilibrium values *ρ*_hot_ = 0 and *T* = *T*_*c*0_. At *t* ~ 150 fs the perturbation reaches the *n* = 15 layer and the dynamics that follows can be neatly divided in three steps.

(i) In the time window 150–400 fs the dynamics is characterized by a significant increase of *ρ*_hot_(15, *t*) highlighting the arrival of the propagating hot-electron population. On this timescale, the electronic relative temperature variation remains limited. This is indicative of a ballistic regime of energy transport in which the energy flows without inducing any heating in the underlying quasi-equilibrium distribution.
(ii) For *t* ≳ 400 fs the hot-electron population displays a sharp drop and, concomitantly, we observe the activation of a fast oscillatory dynamics in the electronic temperature of the cold electrons. Initially the oscillations are centered around a value higher than the initial equilibrium temperature *T*_*c*0_ indicating that the transit of the ballistic front of hot electrons induced the heating of the population of cold electrons on the layer.
(iii) Eventually the system equilibrates for *t* ≳ 0.9 ps with the residual damped temperature oscillations converging to *T*_*c*0_.

We gain further insight into the thermal dynamics by comparing the dynamics of the local cold electronic temperature with the heat flux *q*_*n*_(*t*) at layer *n*. Figure 3b reports the spatial profiles of the heat flux (right axis, blue trace) and of the local electronic temperature (left axis, red trace) at fixed instants of time. The broad feature at the forefront of the heat-flux profile indicates the propagation of a ballistic energy front accompanied by a small and more localized perturbation of the electronic temperature. At the back front of the ballistic heat flux, as indicated by the blurred yellow band in Fig. 3b, we observe the formation of a sharp sinusoidal feature in the spatial profile of the temperature. In the time domain, this sharp feature marks the separation between the first two dynamical regimes of the local temperature observed in panel a for the layer *n* = 15. The presence of this pronounced oscillation of the temperature spatial profile is accompanied by weaker temperature oscillations with smaller spatial periodicity in the layers behind the ballistic front.

To fully characterize the thermal dynamics regimes occurring after the ballistic front has transited, we further compare the dynamics of the heat flux with that of the temperature gradient ∇_⊥_*T*(*t*) = (*T*_*n*+1_(*t*) − *T*_*n*_(*t*))/*a* perpendicular to the layers. These quantities are shown in Fig. 3c for the *n* = 15 layer. In the time window 0.15–0.4 ps, the ballistic regime shows up as a sharp increase of the heat flux with no sizeable effect on the temperature gradient. In correspondence of the end of the ballistic regime, i.e. the sharp drop of the heat current, an oscillatory dynamics is activated for the temperature gradient. The oscillatory dynamics of ∇_⊥_*T*(*t*) is maintained in the 0.4–0.9 ps time window, along with a residual positive heat current on the layer. At *t* ≳ 0.9 ps the heat current displays damped oscillations centred around zero indicating the recovery of local thermal equilibrium. Remarkably, the equilibration is characterized by the synchronization between the dynamics of the temperature gradient and the heat flux. In this regime, we can define an instantaneous proportionality between the heat flux and temperature gradient, i.e. *q*_*n*_(*t*) ∝−∇_⊥_*T*(*t*), indicating that the heat transfer process is well described by a Fourier-like heat transfer law.

At intermediate times (0.4 < *t* < 0.9 ps), before Fourier-like transport sets in, there is a residual positive flow of the heat current with an oscillatory dynamics of −∇_⊥_*T*(*t*) that is not simply proportional to that of *q*_*n*_(*t*). This fact reveals the presence of a new heat transport regime, which bridges the ballistic regime established at the arrival of the perturbation (0.15 < *t* < 0.4 ps) and the Fourier-like transport setting in at long times after the perturbation has transited (*t* > 0.9 ps). This intermediate regime is characterized by a residual population of hot electrons on the layer and by an oscillatory dynamics of the temperature of the cold electron population. We identify this regime as a hydrodynamic transport of heat sustained by the exchange of energy between the two sub-populations of hot and cold electrons. By comparing the dynamics on the single layer (Fig. 3a, c) with the layer profiles at different times (Fig. 3b), we can observe that the emergence of the hydrodynamic regime coincides with transit of the sharp sinusoidal feature in the spatial profile of the temperature at the trailing edge of the heat-flux ballistic front. As it will be further discussed, this feature can be considered as a temperature wave-packet propagating through the system.

Summarizing, the sub-picosecond thermal dynamics of electrons displays three subsequent regimes of heat transport: (i) the ballistic propagation of energy at the front of the perturbation; (ii) the hydrodynamic regime at the trailing edge of the ballistic front. The former is characterized by a wave-like propagation of the electronic temperature; (iii) a Fourier-like heat transport driving the recovery of thermal equilibrium. The time and space extension of the three regimes are indicated by the arrows in the plots of the dynamics at fixed layer index (see Fig. 3c) and of spatial profiles at fixed time (see Fig. 3b). In the remaining of the paper we analyse in detail the different regimes and discuss the possibility to control their onset in layered correlated materials.

### Ballistic energy transport

We now address the possibility of controlling the initial ballistic energy transport by tuning the microscopic parameters entering in the Hubbard model (1). In the ballistic regime, the energy is mostly carried by the population of hot electrons at temperature *T*_*h*_. The energy propagates through hopping processes of the hot electrons excited in the first layers. Layered correlated materials thus offer two complementary ways to control the inter-layer coupling and, in turn, the velocity of propagation of the ballistic front, namely tuning either the anisotropy of the system, *t*_⊥_/*t*_∥_, or the strength of the interaction, *U*. The increase of the latter drives a reduction of the quasiparticle weight *Z*, which leads to a larger effective mass for the inter-layer motion and a smaller effective hopping, ${t}_{\perp }^{* }=Z{t}_{\perp }$.

**Fig. 4:**
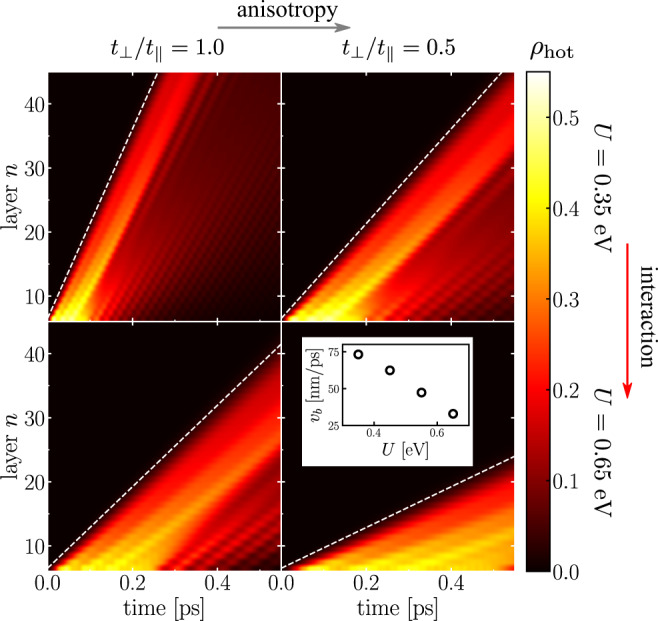
Control of ballistic energy propagation. The four panels matrix displays the ballistic dynamics of the hot-electron population for varying values of the correlation strength *U* and anisotropy *t*_⊥_/*t*_∥_ (*t*_∥_ = 60 meV). The color scale represents the amplitude of *ρ*_hot_(*n*, *t*) (yellow: maximum; black: minimum). The inset displays the speed *v*_b_ of the ballistic wavefront for different values of *U* at *t*_∥_ = *t*_⊥_. The ballistic wavefronts are highlighted by the dashed lines in the main panels.

We show these effects in Fig. 4 where we report the spatio-temporal dynamics of the hot-electron population *ρ*_hot_(*n*, *t*) obtained for different values of anisotropy (horizontal gray arrow) and relative interaction strength (vertical red arrow). Increasing either one, the propagation velocity of the wavefront is diminished. In the inset we plot the velocity of ballistic propagation *v*_b_ as a function of *U* for *t*_⊥_/*t*_∥_ = 1. *v*_b_ is defined as the slope of the white dashed line in Fig. 4. The correlation-induced renormalization of ${t}_{\perp }^{* }$ strongly suppresses the energy propagation along the *z*-direction. For the sake of applications, we note that, in nanosystems with sizes of the order of the ballistic mean free-path, the thermal conductivity becomes a size-dependent property^46–49^. Nanoengineering of LCM, combined with proper tuning of *U* and *t*_⊥_/*t*_∥_, thus offers a mean to control, on the picosecond timescale, the velocity of ballistic heat pulses and, therefore, the thermal conductivity of nanodevices.

### Hydrodynamic energy transport: temperature waves

The results reported in Fig. 3 demonstrate that a purely electronic hydrodynamic transport regime can be achieved in our correlated system on much faster timescales than the more conventional phononic counterpart^13,14,19^. Similarly to the phononic case, this hydrodynamic regime manifests itself by a wave-like propagation of temperature oscillations, which emerge after the ballistic front has transited (see arrows in Fig. 3c). In this section, we will quantitatively describe the characteristics of temperature wave-like propagation, as it emerges from our microscopic model, and compare with a macroscopic model for the description of the hydrodynamic regime characterized by the emergence of electronic temperature waves.

**Fig. 5:**
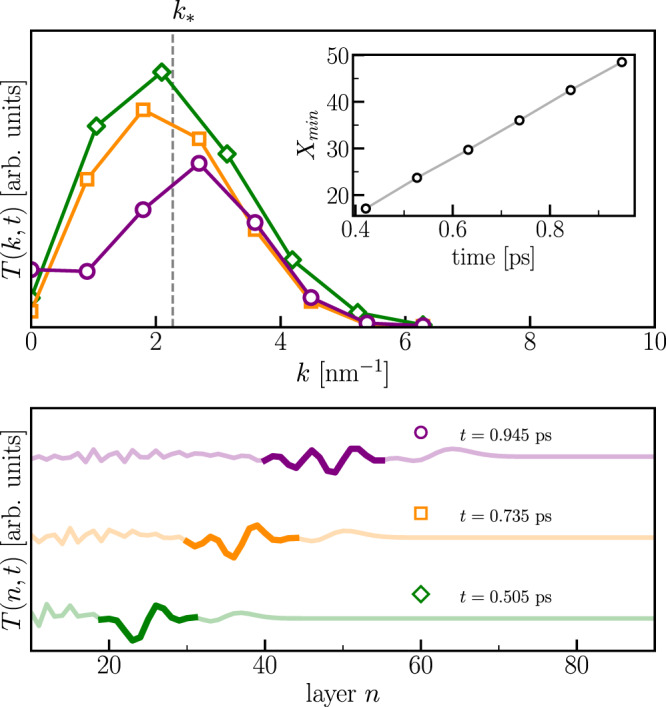
Spectral analysis in **k**-space of the electronic temperature waves. Top panel: spatial Fourier transform of the layer profile of the electronic temperature at three different times, *t* = 0.505 ps (diamonds), *t* = 0.735 ps (squares) and *t* = 0.945 ps (circles). The inset shows the position of the minimum of the temperature oscillation, indicated in the bottom panel, as a function of time. The vertical dashed line indicated by k_*_ shows the average of the position of the three peaks. Bottom panel: Portions of the temperature profiles at different times used to compute the discrete Fourier transform. The arrows indicate the positions *X*_*min*_ and *X*_*max*_.

In the microscopic model, we characterize the hydrodynamic regime by tracking the position of the minimum of the wave packet ${X}_{\min }$. We observe that ${X}_{\min }$ linearly increases in time (See the inset of Fig. 5a), allowing an estimate of the wave-packet group velocity from the simple relation ${X}_{\min }={v}_{g}t$, with *v*_*g*_ ~ 30 nm/ps of the same order of magnitude of the ballistic energy wavefront velocity. A similar result is obtained when tracking the time-dependent maximum of the wave packet, ${X}_{\max }$. This result suggests that we can approximately describe the wave packet as a superposition of weakly dispersive waves with frequencies *ν*_*k*_ = *v*_*g*_*k*/2*π*.

In order to identify the barycentric wavevector of the propagating wave packet, in the top panel of Fig. 5 we report the spatial Fourier transform of the electronic temperature profile in the spatial window where the propagating packet is present, as highlighted in the three curves of the bottom panel, which correspond to three different times, *t* = 0.505 ps, *t* = 0.735 ps and *t* = 0.945 ps. The small number of layers included in the Fourier window produces spectrally broaden peaks with the maximum occurring at slightly different *k* values for different times. We estimate the peak wavevector by taking the average of the three peaks observed at the three chosen times, obtaining k_*_ ~ 2.2 nm^−1^, corresponding to a wavelength *λ* ~ 2.85 nm. Inserting this result in the linear dispersion relation we obtain a frequency ν_*_ ~ 10.5 THz. We notice that in the time domain, and at fixed layer index, this frequency corresponds to the inverse of the period of the large amplitude temperature oscillation originating after the transit of the ballistic energy wavefront, as shown in Fig. 3a.

We now compare the predictions of the microscopic model to a macroscopic description of electronic temperature waves based on the phenomenological Dual Phase Lag Model (DPLM)^50^. The DPLM builds on a modification of Fourier law by the introduction of a delay between the time at which the temperature gradient ∇_⊥_*T* is established, *t* + *τ*_*T*_, and the time when the inter-layer heat-flux *q* sets in, *t* + *τ*_*q*_

7 $$q(t+{\tau }_{q},z)=-{\kappa }_{T,el}\,{\nabla }_{\perp }T\left(t+{\tau }_{T},z\right).$$

This approach, although phenomenological, allows to easily accounts for both thermal wave damping, and dispersion. Recently, it proved effective in describing phononic temperature wave oscillations in graphite^18^.

The expansion of Eq. (7) to first order, and its combination with the local conservation of energy at time *t*, gives rise to a second order parabolic differential equation for the temperature variation Δ*T*(*t*, *z*) = *T*(*t*, *z*) − *T*_c0_. We look for wave-like solutions of this differential equation starting from a temperature pulse triggered at initial time on the top side of the sample slab. Following ref. ^18^, the pulse can be described by a superposition of plane-waves of real-valued wave vectors *k* and complex frequencies *ν*. Underdamped plane-wave solutions for Δ*T*(*t*, *z*) are found if the condition *τ*_*q*_ > 2*τ*_*T*_ is met. These temperature waves are characterized by the complex-valued dispersion relation

8 $$\nu (k,R,\alpha )={\nu }_{1}(k,R,\alpha )+{{{{{{{\rm{i}}}}}}}}{\nu }_{2}(k,R,\alpha ),$$

where *ν*_1,2_(*k*, *R*, *α*) depend on the wavevector *k* and on the parameters $R=\frac{{\tau }_{T}}{{\tau }_{q}}$ and thermal diffusivity $\alpha =\frac{{\kappa }_{T,el}}{{C}_{el}}$, *κ*_*T*,*el*_ and *C*_*el*_ being the electronic thermal conductivity and specific heat, respectively. The analytical expressions for the real-valued *ν*_1_ and *ν*_2_, together with the quality factor defined as $Q(k,R,\alpha )=\frac{{\nu }_{1}}{{\nu }_{2}}$, are reported in the Supplementary Eqs. (1)–(3). It is important to stress that the derivation of the dispersion for the DPLM, Eq. (8), directly follows from energy conservation and the assumption of a delay between the formation of a gradient of temperature and the heat flux. As such, there is no direct connection between the DPLM and the assumptions used for studying the thermal dynamics in the microscopic model. We further notice that, in principle, the quantities *R* and *α* do depend on the electronic temperature *T*. However, since we are looking for relative variation ∣Δ*T*(*t*)∣/*T*_*c*0_ ≪ 1 (see Fig. 3a), the temperature dependence may be limited to the initial base temperature *T*_*c*0_, i.e. *R* = *R*(*T*_*c*0_) and *α* = *α*(*T*_*c*0_).

In order to reveal under which conditions temperature waves are sustained, we exploit the dispersion *ν*_1_(*k*) and its quality factor *Q*, upon inserting in Eq. (8) the parameters relevant for SrVO_3_. In particular, we extract the thermodynamic quantities from the literature, whereas we use the sub-picosecond dynamics of the microscopic model to estimate the delay time τ_q_. We first identify the time for setting a variation in the temperature gradient, *τ*_*T*_, as the electronic thermalization time. The local thermalization time in SVO is estimated to be as short as ~5 fs on the basis of angle-resolved photoemission spectroscopy^51^ and optical conductivity^36^ data (see Supplementary Fig. 1). This timescale is compatible with the attribution in the microscopic model of an instantaneous local temperature on the sub-picosecond timescale, as described in Fig. 3. We thus set *τ*_*T*_ = 5 fs. On the other hand, the heat-flux dynamics in Fig. 3c shows that the synchronization between ∇_⊥_*T* and *q* starts at ~900 fs, i.e. 500 fs after the ballistic wavefront has transited through the 15th layer. We can thus assume *τ*_*q*_ ≃ 500 fs. Based on these assumptions, we obtain *R* = *τ*_*T*_/*τ*_*q*_ ~ 0.01, which is well below the threshold *R* < 0.5 for the observation of a wave-like behaviour^18^. While the electronic scattering time is expected to weakly depend on the temperature, the temperature dependence of *τ*_*q*_ is tested by studying the dynamics of the single-band Hubbard model at different base temperatures *T*_*c*0_. As shown in Supplementary Fig. 2, *τ*_*q*_ is almost independent of *T*_*c*0_, thus allowing to assume a temperature independent value of *R*. The temperature dependence of the wave frequencies is instead retained through the thermal diffusivity *α*. Specifically, for the case of SVO, *C*_*el*_ = *γT* with *γ* = 2.4 × 10^2^ Jm^−3^ K^−2^ ^52^. As for *κ*_*T*,*el*_(*T*) we retrieve it from the temperature-dependent electrical conductivity, *σ*(*T*), of SVO single crystals^52^ upon application of the Wiedemann-Franz-Lorentz relation: *κ*_*T*,*el*_ = *LσT*, *L* = 2.44 ⋅ 10^−8^ WΩK^−2^ being the Lorentz number. The temperature-dependent *κ*_*T*,*el*_ ranges from ≃10 Wm^−1^K^−1^ at 300 K to ≃20 Wm^−1^K^−1^ at 35 K.

**Fig. 6:**
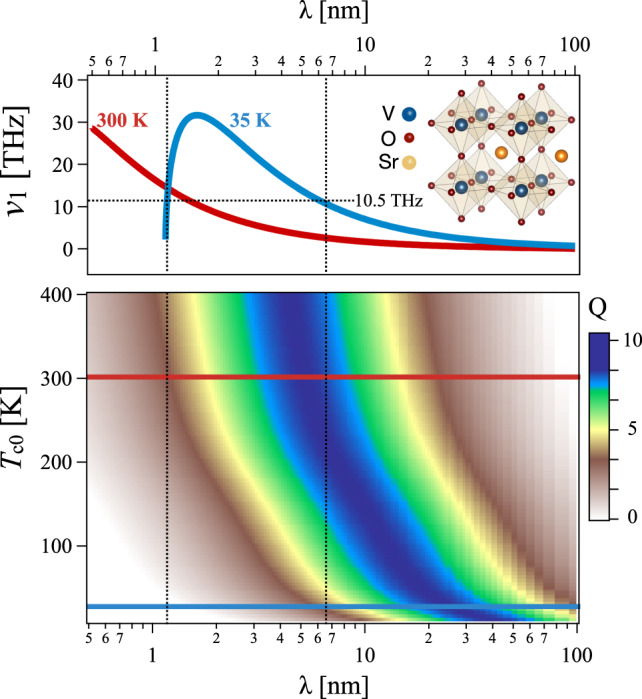
Temperature wave dispersion in SVO_3_. Top panel: electronic temperature oscillation frequency vs oscillation’s wavelength, *λ*, at a base temperatures *T*_*c*0_ = 35 K (blue line) and 300 K (red line). Inset: Structure of SVO_3_. Bottom panel: quality factor (colormap) as a function of the oscillation’s wavelength, *λ*, and the base temperature *T*_*c*0_. Calculations are based on the dispersion relations, Supplementary Eqs. (1)–(3), upon insertion of input parameters from experiments, *α* and *τ*_*T*_, and from non-equilibrium thermal dynamics results from the layered Hubbard model (see text), *τ*_*q*_. In both panels a linear-log plot is adopted.

With this parameters at hand, in Fig. 6 we show the dispersion relation for the temperature oscillation frequency *ν*_1_ (top panel) and the corresponding *Q*-factor (bottom panel) as a function of wavelength *λ* = 2*π*/*k* and base temperature *T*_*c*0_. The temperature wave frequency *ν*_∗_ ~ 10.5 THz, obtained from the microscopic model at the base temperature *T*_*c*0_ = 35 K, falls within the range of the allowed frequencies and is compatible with two possible wavelengths, *λ* ~ 6.5 nm and *λ* ~ 1.1 nm. These wavelengths correspond to *Q*-factors ~5 and 0.2, respectively, therefore only the longest wavelength is expected to be detectable. This wavelength falls pretty close to the estimate *λ* ~ 2.85 nm obtained from the microscopic single-band Hubbard model.

Given the quite general assumptions on the parameters of the microscopic model and the realistic values used in the phenomenological model, the above comparison shows an overall good agreement between the temperature waves dynamics obtained from the sub-picoseconds dynamics of the single-band Hubbard model and the predictions based on a macroscopic model. Such an agreement further confirms that LCM can sustain, in the hydrodynamic regime, temperature waves with wavelengths and periods fully compatible with state-of-the-art materials growth techniques and time-resolved spectroscopies. More in general, the frequencies and *Q*-factor values reported in Fig. 6 show that the manifestation of temperature waves in LCM can be observed up to temperatures as high as 300 K. This is the consequence of the fact that the energy scales controlling the electronic dynamics, i.e. *t*_∥_ = *t*_⊥_ = 60 meV and *U* = 650 meV, correspond to temperatures of ≃700 K and ≃7000 K, respectively. At variance with the phononic case, the sub-picosecond electronic hydrodynamic regime is thus expected to be very robust against temperature, giving rise to the emergence of temperature wave-like oscillations in real materials at ambient conditions.

**Fig. 7:**
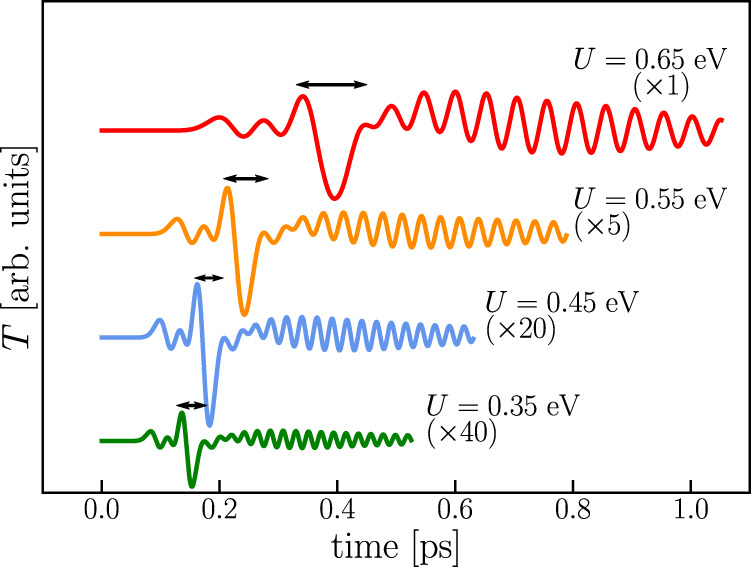
Control of temperature wave-like oscillations. Temperature *T*(*t*) of the “cold” electronic oscillation at the *n* = 15 layer for different values of *U* (the same values used in the inset of Fig. 4) Some of the data have been magnified and the curves shifted for graphical reasons. The horizontal arrows highlight the oscillation periods that match the frequency ν_*_ extracted from spectral analysis of the temperature wave packet.

Similarly to the ballistic transport regime, the electronic correlations are key to control the wave-like temperature propagation. In Fig. 7 we report the temperature dynamics at layer *n* = 15 for different values of the interaction *U*. We observe that the smaller the interaction the smaller is the temperature oscillation amplitude triggered in the population of cold electrons by the transit of the hot-electron wavefront. The data further show that the temperature oscillation periods, indicated in Fig. 7 by the black arrows, decrease as the interaction *U* is decreased. In general, as already observed for the case of the ballistic energy propagation, the thermal dynamics of quasiparticles becomes slower as the interaction is increased. This may be traced back to the effect of the correlation-driven renormalization of the quasiparticle effective mass. Therefore, the electronic correlations strength, which controls the quasiparticle effective mass renormalization, can act as a control parameter for the frequency and amplitude of transient temperature waves in LCM.

### Recovery of Fourier-like heat transport

After the transit of the ballistic heat wavefront and of the temperature wave packet, the hydrodynamic regime gradually evolves into a more conventional dynamics (*t* > 0.9 ps in Fig. 3). Here, the non-equilibrium hot-electron population has already left the region of interest, giving rise to a free oscillatory equilibration dynamics of the temperature of “cold" electrons. The wavelength of the temperature oscillation is smaller than that of the temperature wave-packet propagating with speed *v*_*g*_ (hydrodynamic regime), as may be seen in Fig. 3b. In the present regime, the oscillation frequency *ν* gives an intuitive physical picture of the temperature oscillations. Indeed, the oscillation frequency *ν* corresponds to an energy *hν*, which exactly matches the energy $4{t}_{\perp }^{* }$ corresponding to the renormalized bandwidth in the direction perpendicular to the layers (Fig. 1). This fact clearly highlights that the oscillatory dynamics of the temperature is governed by the particle inter-layer hoppings between occupied and empty states due to unbalanced energy distributions on the different layers. In this simple picture, the heat flux left behind by the temperature wave packet freely oscillates with a frequency controlled by ${t}_{\perp }^{* }$, which is the only intrinsic energy scale of the Hubbard Hamiltonian playing a role on the hundreds femtoseconds timescale. The oscillating *q*_*n*_(*t*) thus acts as the source for the temperature gradient, which instantaneously follows the temperature variation, i.e. without any delay, as expressed in Fourier law $q\left(t,z\right)=-{\kappa }_{T,el}\,{\nabla }_{\perp }T\left(t,z\right)$. For instance, for *U* = 0.65 eV one has ${t}_{\perp }^{* }\simeq 0.33{t}_{\perp }=20\,{{{{{{{\rm{meV}}}}}}}}$ and the oscillation periods reads $h/4{t}_{\perp }^{* }\simeq 50\,{{{{{{{\rm{fs}}}}}}}}$.

Interestingly, we can estimate the electronic thermal conductivity by evaluating the ratio between the oscillation amplitude of the heat flux and that of the temperature gradient, i.e. *κ*_*T*,*el*_ = ∥*q*∥/∥∇_⊥_*T*∥ where the symbol ∥…∥ represents the oscillation amplitude. As an example of the moderately interacting regime we consider *U* = 0.45 eV, resulting in $\frac{U}{{U}_{c}} \sim 0.55$, that is quite far from the Mott transition critical point, *U*/*U*_*c*_ ~ 1. In doing so we estimate *κ*_*T*,*el*_ ≃ 440 Wm^−1^ K^−1^, which is in the order of typical zero-frequency electronic thermal conductivity of conventional metals. On the other hand, when we increase *U*, the interactions drive a larger temperature gradient (see Supplementary Fig. 3), which in turn results in a very small value of *κ*_*T*,*el*_^53^. For instance, when *U*/*U*_*c*_ = 0.7–0.8, the estimated thermal conductivity is in the range 40–2.5 Wm^−1^ K^−1^, a value of the same order of the zero-frequency conductivity reported for SVO^52^. Thus, despite its simplicity, the thermal dynamics of the layered Hubbard model predicts the correct order of magnitude of Fourier-like thermal conductivity of materials for a very wide range of correlation strengths.

In conclusion, we have proposed layered correlated materials as a platform enabling to access a rich variety of heat transport regimes. We have considered a layered Hubbard model and studied the thermal dynamics triggered by the creation of a non-equilibrium population of hot electrons on the top of the system. The transient dynamics undergoes, on ultrashort space and timescales, a crossover between ballistic energy transport and electronic temperature wave-like oscillations in the hydrodynamic regime. Eventually, the Fourier-like heat transport regime is recovered on the picosecond timescale. Specifically, transition metal oxides thin films and heterostructures, with typical thicknesses and periodicities in the few nanometers range, are here predicted to sustain electronic temperature wave-like oscillations in a parameter space fully compatible with state-of-the-art time-resolved calorimetry techniques^54^. We stress that, in contrast to the phononic case for which scattering is detrimental to the formation of temperature waves, in the present electronic case the correlation strengths are the key ingredients to observe this phenomenon. As a results, electronic temperature waves can exists for a much broader range of temperatures, and are predicted to persist up to room temperature.

The outreach of our results ranges beyond LCM. Among the most interesting applications we foresee is nanoengineering of superlattices made out of correlated materials, allowing for coherent control of temperature waves in nanodevices. For instance, LCM can be grown in heterostructures with control of the physical properties at the level of single atomic layers^55^ and with the possibility of engineering artificial periodicities to select high-*Q* modes of temperature waves. The recent introduction of the temperonic crystal^21^, i.e. a periodically modulated structure, which behaves like a crystal for temperature waves, provides a new tool to coherently control temperature pulses in correlated heterostructures. Furthermore, strong correlations, and their control via the inter-layer twist angle, have recently been reported in graphene superlattices^56–58^. The present work paves the way to the control of electronic ballistic propagation and to the engineering of nanodevices exploiting the wave-like nature of the electronic heat transfer on the sub-picosecond timescale.

## Methods

### Gutzwiller variational dynamics

In this section we describe the general scheme of the Gutzwiller variational dynamics used in this work to address the thermal dynamics in the layered Hubbard model. We refer the reader to existing literature for an in-depth review about the method^41,42,44^. We start from the definition of a variational density matrix of the form

9 $$\hat{\rho }(t)\equiv {{{{{{{\mathcal{P}}}}}}}}(t){\hat{\rho }}_{* }(t){{{{{{{{\mathcal{P}}}}}}}}}^{{{{\dagger}}} }(t),$$

where ${\hat{\rho }}_{* }(t)$ represents the density matrix of an effective system of non-interacting quasiparticles, and ${{{{{{{\mathcal{P}}}}}}}}(t)={\prod }_{in}{{{{{{{{\mathcal{P}}}}}}}}}_{in}(t)$ is a projector, defined by operators ${{{{{{{{\mathcal{P}}}}}}}}}_{in}$ acting on the local Hilbert spaces defined at the site with in-plane index *i* and layer index *n*. The projectors ${{{{{{{{\mathcal{P}}}}}}}}}_{in}(t)$ control the relative weights of the local many-body configurations. We obtain dynamical equations of motions by using the Dirac-Frenkel time-variational principle applied to the Gutzwiller ansatz (9). This variational dynamics has been originally introduced for the description of the dynamics of pure quantum states^41^, whereas extension to the dynamics of mixed states has been presented in ref. ^44^. By representing the local projectors by a set of matrices ${\hat{{{\Phi }}}}_{in}$ defined on the local Hilbert space, the variational dynamics is defined by the set of coupled equation of motions

10 $$i\hslash {\partial }_{t}{\hat{\rho }}_{* }(t)=\left[{H}_{* }[\hat{{{\Phi }}}(t)],{\hat{\rho }}_{* }(t)\right]$$

11 $$i\hslash {\partial }_{t}{\hat{{{\Phi }}}}_{in}(t)=\frac{\delta }{\delta {{{\Phi }}}_{in}^{{{{\dagger}}} }(t)}{{{{{{{\rm{Tr}}}}}}}}\left[{\hat{\rho }}_{* }(t){H}_{* }[\hat{{{\Phi }}}(t)]\right]+{H}_{in}{\hat{{{\Phi }}}}_{in}(t).$$

In the above equations ${H}_{* }[\hat{{{\Phi }}}(t)]$ is a single-particle Hamiltoinan obtained by replacing the fermionic operators in the hopping Hamiltonian *H*_0_, ${c}_{in\sigma }^{{{{\dagger}}} }\to {R}_{in}[\hat{{{\Phi }}}]{c}_{in\sigma }^{{{{\dagger}}} }$, with ${R}_{in}[\hat{{{\Phi }}}]$ hopping renormalization parameters determined by the local projectors ${\hat{{{\Phi }}}}_{in}$. *H*_*in*_ is the matrix representation of the Hubbard interaction onto the local Hilbert space. The density matrix ${\hat{\rho }}_{* }(t)$ describes the dynamics of effective quasiparticles with renormalized hopping or, equivalently, enhanced mass. The quasiparticle renormalization is defined as ${Z}_{i}=| {R}_{i}[\hat{{{\Phi }}}]{| }^{2}\ \le \ 1,$ and it is controlled by the local projectors ${\hat{{{\Phi }}}}_{i}$. The local projectors ${\hat{{{\Phi }}}}_{i}$ describe the dynamics of the local degrees of freedom associated with the interaction term. In the single-band case at half-filling, the local degrees of freedom reduce to the excitations of double occupied sites (doublons) at energies ~*U*. The two dynamics are coupled so that the method is able to capture a non-trivial feedback between the delocalized (quasipartcles) and localized (doublons) degrees of freedom. It is important to mention that Eqs. ((10)–(11)) represent an exact solution of the variational problem only in the limit of a lattice of infinite coordination. For lattices of finite coordination Eqs. ((10)–(11)) represent an approximation to the variation problem, known as the Gutzwiller approximation. In this work, we consider full in-plane translation invariance, so that the matrices Φ_*in*_ depend only on the layer index *n*. The non-equilibrium dynamics is completely determined by the solution of the set dynamical Eqs. ((10)–(11)) with initial conditions set by the non-equilibration protocol described below.

### Non-equilibrium protocol

The non-equilibrium protocol defines the initial condition for the unitary dynamics described in this work. We first solve the variational problem at *T* = 0, by looking at stationary solutions of Eqs. ((10)–(11)) for a pure quantum state ${\hat{\rho }}_{* }=\left|{{{\Psi }}}_{* }(t)\right\rangle \left\langle {{{\Psi }}}_{* }(t)\right|$. Therefore, we set the temperatures of the two-electronic populations by running an equilibration dynamics in which each layer is coupled to an external bath at temperature *T*_*n*_. To this extent we supplement the unitary dynamics for the quasiparticles (10) with a dissipative term ${{{{{{{{\mathcal{L}}}}}}}}}_{{{{{{{{\rm{bath}}}}}}}}}$

12 $$i\frac{\partial {\rho }_{* }(t)}{\partial t}=\left[{H}_{* }(t),{\rho }_{* }(t)\right]+{{{{{{{{\mathcal{L}}}}}}}}}_{{{{{{\mathrm{bath}}}}}}}[{\rho }_{* }].$$

which is obtained by considering, on each layer, a non-interacting bath defined on the same square lattice of the Hubbard layer. We assume all the baths to be identical, so that the bath Hamiltonian reads ${H}_{{{{{{{{\rm{bath}}}}}}}}}={\sum }_{{{{{{{{\bf{k}}}}}}}}n\sigma }{\epsilon }_{bath}({{{{{{{\bf{k}}}}}}}}){d}_{{{{{{{{\bf{k}}}}}}}}n\sigma }^{{{{\dagger}}} }{d}_{{{{{{{{\bf{k}}}}}}}}n\sigma }^{}$. The interaction with the electrons in the layers is described by local single-particle hopping processes ${H}_{{{{{{{{\rm{bath-sys}}}}}}}}}=v{\sum }_{in\sigma }{c}_{in\sigma }^{{{{\dagger}}} }{d}_{in\sigma }^{}+h.c.=v{\sum }_{{{{{{{{\bf{k}}}}}}}}n\sigma }{c}_{{{{{{{{\bf{k}}}}}}}}n\sigma }^{{{{\dagger}}} }{d}_{{{{{{{{\bf{k}}}}}}}}n\sigma }^{}+h.c.$. By integrating out the baths in the so-called Born-Markov approximation, and by assuming that the baths belonging to different layers are completely decoupled, i.e. $\langle {d}_{{{{{{{{\bf{k}}}}}}}}n\sigma }^{{{{\dagger}}} }{d}_{{{{{{{{\bf{k}}}}}}}}n^{\prime} \sigma }^{}\rangle \propto {\delta }_{nn^{\prime} }$, we obtain a standard Lindbladt form of the dissipative term containing both single-particle losses, ${{{{{{{{\mathcal{L}}}}}}}}}^{-}$, and gains, ${{{{{{{{\mathcal{L}}}}}}}}}^{+}$,

13 $${{{{{{{{\mathcal{L}}}}}}}}}_{{{{{{\mathrm{bath}}}}}}}[{\rho }_{* }]={{{{{{{{\mathcal{L}}}}}}}}}^{+}[{\rho }_{* }]+{{{{{{{{\mathcal{L}}}}}}}}}^{-}[{\rho }_{* }],$$

with

14 $${{{{{{{{\mathcal{L}}}}}}}}}^{-}[{\rho }_{* }]=\mathop{\sum}\limits_{{{{{{{{\bf{k}}}}}}}}n\sigma }{{{\Gamma }}}_{{{{{{{{\bf{k}}}}}}}}n}^{-}(t)\left[2{c}_{{{{{{{{\bf{k}}}}}}}}n\sigma }^{}{\rho }_{* }(t){c}_{{{{{{{{\bf{k}}}}}}}}n\sigma }^{{{{\dagger}}} }-\{{c}_{{{{{{{{\bf{k}}}}}}}}n\sigma }^{{{{\dagger}}} }{c}_{{{{{{{{\bf{k}}}}}}}}n\sigma }^{},{\rho }_{* }(t)\}\right]$$

15 $${{{{{{{{\mathcal{L}}}}}}}}}^{+}[{\rho }_{* }]=\mathop{\sum}\limits_{{{{{{{{\bf{k}}}}}}}}n\sigma }{{{\Gamma }}}_{{{{{{{{\bf{k}}}}}}}}n}^{+}(t)\left[2{c}_{{{{{{{{\bf{k}}}}}}}}n\sigma }^{{{{\dagger}}} }{\rho }_{* }(t){c}_{{{{{{{{\bf{k}}}}}}}}n\sigma }^{}-\{{c}_{{{{{{{{\bf{k}}}}}}}}n\sigma }^{}{c}_{{{{{{{{\bf{k}}}}}}}}n\sigma }^{{{{\dagger}}} },{\rho }_{* }(t)\}\right].$$

The couplings ${{{\Gamma }}}_{{{{{{{{\bf{k}}}}}}}}}^{\pm }(t)$ are defined by the distribution functions of the baths as

16 $${{{\Gamma }}}_{{{{{{{{\bf{k}}}}}}}}n}^{+}\equiv r(t){{\Gamma }}{N}_{n}^{{{{{{\mathrm{bath}}}}}}}({{{{{{{\bf{k}}}}}}}})\,\qquad {{{\Gamma }}}_{{{{{{{{\bf{k}}}}}}}}n}^{-}\equiv r(t){{\Gamma }}(1-{N}_{n}^{{{{{{\mathrm{bath}}}}}}}({{{{{{{\bf{k}}}}}}}})),$$

where Γ is a constant proportionals to the couplings between layer and baths Γ ∝ *v*^2^ and *r*(*t*) = *θ*(*t* − *t*_−*∞*_)*θ*(− *t*) is a box function function highlighting that the coupling is switched-on at a time *t*_−*∞*_ < 0 and switched-off at time *t* = 0 when the equilibration is reached. ${N}_{n}^{{{{{{\mathrm{bath}}}}}}}({{{{{{{\bf{k}}}}}}}})=f({\epsilon }_{{{{{{\mathrm{bath}}}}}}}({{{{{{{\bf{k}}}}}}}}),{T}_{{{{{{\mathrm{bath}}}}}}}(n))$ is the Fermi-distribution of the *n*th bath at temperature *T*_*bath*_(*n*). The structure of the couplings reflects the fact that the probability of jumping from the bath to the layer (gain) in a given momentum state **k** is proportional to the occupation number in the corresponding state in the bath. On the contrary, the probability to jump from the layer to the bath (loss), is proportional to the probability of finding an empty state in the bath. The Eq. (12) is solved together with the coupled equation for the local degrees of freedom (11), so that the coupling of quasiparticles with the baths has an indirect effect also onto the local degrees of freedom and, in turn, on the quasiparticle renormalization *Z*. The quasiparticle renormalization corresponding to the mass enhancement parameter mentioned in the main text *m*/m* ≃ 0.3 corresponds to the value obtained at the end of the equilibration step. The absolute value ∣*t*_−*∞*_∣ corresponds to the time needed to reach equilibration, being larger the smaller the couplings Γ. In practice, it is found that the stationary solution does not depend on the strength of the coupling, so that the equilibration can be reached considering an equilibration dynamics much shorter than the picosecond dynamics discussed in the main text.

When equilibration is reached, the density matrix $\hat{\rho }(t)$ approaches a stationary value ${\hat{\rho }}_{{{{{{{{\rm{equ}}}}}}}}}\equiv {{{{{{{{\mathcal{P}}}}}}}}}_{{{{{{{{\rm{equ}}}}}}}}}{\hat{\rho }}_{* }^{{{{{{{{\rm{equ}}}}}}}}}{{{{{{{{\mathcal{P}}}}}}}}}_{{{{{{{{\rm{equ}}}}}}}}}^{{{{\dagger}}} }$, independent of time. In the equilibrated state, the quasiparticle occupation numbers ${N}_{n}^{{{{{{{{\rm{equ}}}}}}}}}({{{{{{{\bf{k}}}}}}}})\equiv {{{{{{{\rm{Tr}}}}}}}}[{\hat{\rho }}_{* }^{{{{{{{{\rm{equ}}}}}}}}}{c}_{{{{{{{{\bf{k}}}}}}}}n\sigma }^{{{{\dagger}}} }{c}_{{{{{{{{\bf{k}}}}}}}}n\sigma }^{}]$ become equal to the occupation numbers of the baths. By changing variable from the quasi-momentum **k** to energy using the bath dispersions *ϵ*_*bath*_(**k**), the equilibration condition, in terms of the quasiparticle energy distribution function, reads

17 $${N}_{n}^{{{{{{{{\rm{equ}}}}}}}}}(\epsilon )\equiv {N}_{n}^{{{{{{{{\rm{equ}}}}}}}}}({\epsilon }_{{{{{{\mathrm{bath}}}}}}}({{{{{{{\bf{k}}}}}}}}))=f(\epsilon ,{T}_{n}).$$

In Fig. 1 we show two energy distribution functions for a surface (*n* ≤ 5), and a bulk (*n* > 5) layer, respectively, obtained after the equilibration step.

### Non-equilibrium distribution functions

Here, we describe the definition of the non-equilibrium distribution functions ${N}_{n}^{{{{{{{{\rm{neq}}}}}}}}}(\epsilon ,t)$. We consider the time evolution of the layer-dependent quasiparticle occupation numbers

18 $${N}_{n}({{{{{{{\bf{k}}}}}}}},t)\equiv {{{{{{{\rm{Tr}}}}}}}}\left[{\hat{\rho }}_{* }(t){c}_{{{{{{{{\bf{k}}}}}}}}n\sigma }^{{{{\dagger}}} }{c}_{{{{{{{{\bf{k}}}}}}}}n\sigma }^{}\right].$$

In order to track the evolution of the occupation numbers with respect to the initial condition (17), we compare *N*_*n*_(**k**, *t*) with the occupation numbers that would be obtained after an equilibration step with a bath at fixed temperature. To this extent, we define non-equilibrium distribution functions from the layer-dependent occupation numbers, Eq. (18), in the same way as done in Eq. (17) for the equilibrated distribution functions, namely

19 $${N}_{n}^{{{{{{{{\rm{neq}}}}}}}}}(\epsilon ,t)\equiv {N}_{n}^{{{{{{{{\rm{neq}}}}}}}}}({\epsilon }_{{{{{{\mathrm{bath}}}}}}}({{{{{{{\bf{k}}}}}}}}),t).$$

We mention that such a definition of a non-equilibrium distribution is needed as the Gutzwiller method does not give direct access to spectral quantities, but only to quantities integrated in frequency. However, the definition (19) is physically motivated by the need of having a meaningful comparison with the distribution functions after the equilibration step, Eq. (17) which are known, being entirely determined by the baths. In particular, at *t* = 0, the definition Eq. (19) correctly reproduces ${N}_{n}^{{{{{{{{\rm{neq}}}}}}}}}(\epsilon ,t=0)={N}_{n}^{{{{{{{{\rm{equ}}}}}}}}}(\epsilon )$, with ${N}_{n}^{{{{{{{{\rm{equ}}}}}}}}}(\epsilon )$ defined in Eq. (17), whereas at *t* > 0, when all the couplings with the baths are set to zero, the relation Eq. (17) is no more satisfied and ${N}_{n}^{{{{{{{{\rm{neq}}}}}}}}}(\epsilon ,t)$ can be expressed as a superposition of two equilibrium Fermi distributions, Eq. (4). This allows for the description of the thermal dynamics in terms of a propagating hot electronic population and the temperature dynamics of the cold electronic population.

## Supplementary information

Supplementary Information

## Data Availability

The data generated in this study, including the code used to generate the data, have been deposited in the YARETA database^59^.
