# Supplementary material for: Thermal dynamics and electronic temperature waves in layered correlated materials
Source: Nat Commun. 2021 Nov 25;12:6904. doi: 10.1038/s41467-021-27081-2 (PMC8616949; doi:10.1038/s41467-021-27081-2)
Supplement: Supplementary file 1 — Supplementary Information [file 41467_2021_27081_MOESM1_ESM.pdf]

Supplementary Information for:
Thermal dynamics and electronic temperature waves in layered correlated materials

Giacomo Mazza,[1] Marco Gandolfi,[2,3] Massimo Capone,[4] Francesco Banfi,[5] and Claudio Giannetti[6,7]

[1] *Department of Quantum Matter Physics, University of Geneva, Quai Ernest-Ansermet 24, 1211 Geneva, Switzerland*
[2] *CNR-INO, Via Branze 45, 25123 Brescia, Italy*
[3] *Department of Information Engineering, University of Brescia, Via Branze 38, 25123 Brescia, Italy*
[4] *CNR-IOM Democritos National Simulation Center and Scuola Internazionale Superiore di Studi Avanzati (SISSA), Via Bonomea 265, 34136 Trieste, Italy*
[5] *FemtoNanoOptics group, Université de Lyon, CNRS, Université Claude Bernard Lyon 1, Institut Lumière Matière, F-69622 Villeurbanne, France*
[6] *Dipartimento di Matematica e Fisica, Università Cattolica del Sacro Cuore, Via Musei 41, I-25121 Brescia, Italy*
[7] *Interdisciplinary Laboratories for Advanced Materials Physics (I-LAMP), Università Cattolica del Sacro Cuore, Via Musei 41, I-25121 Brescia, Italy*

ALLOWED FREQUENCIES AND WAVEVECTORS

As discussed in Ref. 1, the wave equation for the temperature variation $\Delta T(t,z)$=$T(t,z)$-T_{c0} is derived from the combination of Eq. 9 and the local energy conservation. A wave-like behaviour of $\Delta T(x,t)$ emerges[1] when the wavevector k falls in the range $k_{lo} < k < k_{hi}$ with

$$k_{lo(hi)} = \sqrt{\frac{2}{\alpha\tau_T}\left(\frac{\tau_q}{\tau_T}\right)\left(1-\frac{1}{2}\frac{\tau_T}{\tau_q}-(+)\sqrt{1-\frac{\tau_T}{\tau_q}}\right)} \tag{1}$$

yielding real and imaginary frequency components:

$$\omega_1 = \mp\sqrt{-\left[\frac{\alpha^2}{4}\left(\frac{\tau_T}{\tau_q}\right)^2 k^4 + \frac{\alpha}{\tau_q}\left[\frac{1}{2}\left(\frac{\tau_T}{\tau_q}\right)-1\right]k^2+\frac{1}{4\tau_q^2}\right]} \tag{2}$$

$$\omega_2 = \left[\frac{1}{2\tau_q}+\frac{\alpha}{2}\left(\frac{\tau_T}{\tau_q}\right)k^2\right] \tag{3}$$

The Q-factor is easily calculated as Q=$|\omega_1|/\omega_2$

SCATTERING TIME IN SVO FROM OPTICAL DATA

The electronic scattering rate can be extracted from optical spectroscopy data. For strongly interacting electrons, the interactions strongly affects the scattering rate and give rise to a frequency dependent scattering rate, which is accounted for by the extended Drude model. The inverse scattering is directly related to the Drude dielectric function through the relation:

$$\frac{1}{\tau(\omega)} = -\frac{\omega_p^2}{\omega}\mathrm{Im}\frac{1}{\epsilon_D(\omega)-\epsilon_\infty} \tag{4}$$

ω_p being the undressed plasma frequency and ϵ_∞ the effective permittivity that accounts for the interband transitions involving electronic states in the valence and other bands. In Fig. 1 we report the optical scattering rate extracted from the optical data reported in Ref. 2. Considering the density of carriers in SVO, n=1.76·10^{22} cm$^{?3}$, we obtain ω_p=4.6 eV, which corresponds to the plasma frequency obtained from the fitting with a Drude model. The calculated $\tau(\omega)$ shows a pronounced decrease in the range 0.1-0.6 eV, which is connected to the strong scattering with modes at very high frequency, in agreement with the photoemission data discussed in the next session. A value $\tau \approx$2 fs is reached at $\omega >$0.6 eV, whereas the low-frequency limit is $\tau \approx$5 fs. If we consider a pump-probe experiment, which represents a promising configuration to observe wave-like temperature oscillations, it is natural to assume that the high-energy (>0.6 eV) excitations, directly photoinjected by the pump pulse (usually in the near-infrared or visible), scatter with

high-energy modes within ≈1 fs. The low-energy electrons generated during this fast relaxation stage experience a progressively slower scattering time, which reaches a constant value of ≈5 fs at energies smaller than 0.1 eV (see Fig. 1). Since thermalization is related to the low-energy carriers, we can assume that the typical thermalization time is τ_T ≈5 fs.

SCATTERING TIME IN SVO FROM PHOTOEMISSION

In order to evaluate the local thermalization time we can also refer to the SVO band structure, as measured by angle-resolved photoemission spectroscopy[3]. The electronic correlations manifest themselves in a high-energy contribution to the electronic self-energy, which exhibits a broad kink at $\hbar\Omega_k$ ∼0.3 eV. We can thus assume that the typical timescale of electron-electron interactions is $1/\hbar\Omega_k$ ∼2 fs, which is perfect agreement with the high-energy scattering rate extracted from optical data, as discussed in the previous section.

SUPPLEMENTARY FIGURES

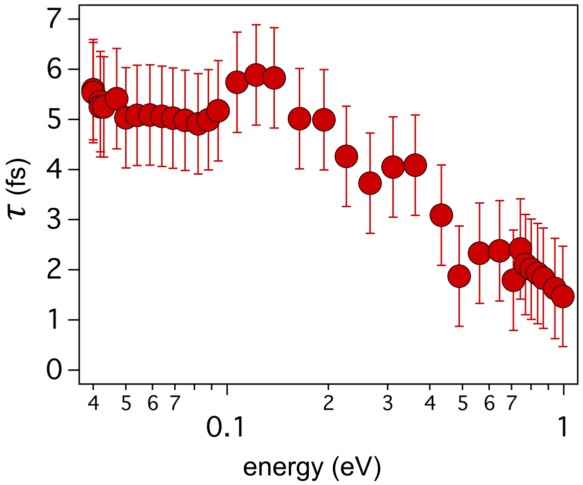

Supplementary Figure 1: Optical scattering time of the conduction electrons for SVO, as extracted from the optical spectroscopy data reported in Ref.[2] by using Eq. 4. The error bars are associated to the uncertainty in extracting the Drude component $\epsilon_D(\omega)$ from the total dielectric function.

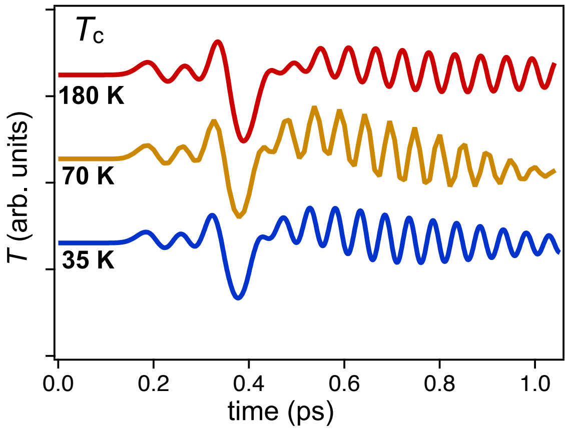

Supplementary Figure 2: Temperature $T(t)$ of the quasi-equilibrium electronic distribution calculated at the 15th layer for different values of the initial base temperature T_c=35 K (red), 70 K (yellow), 180 K (red).

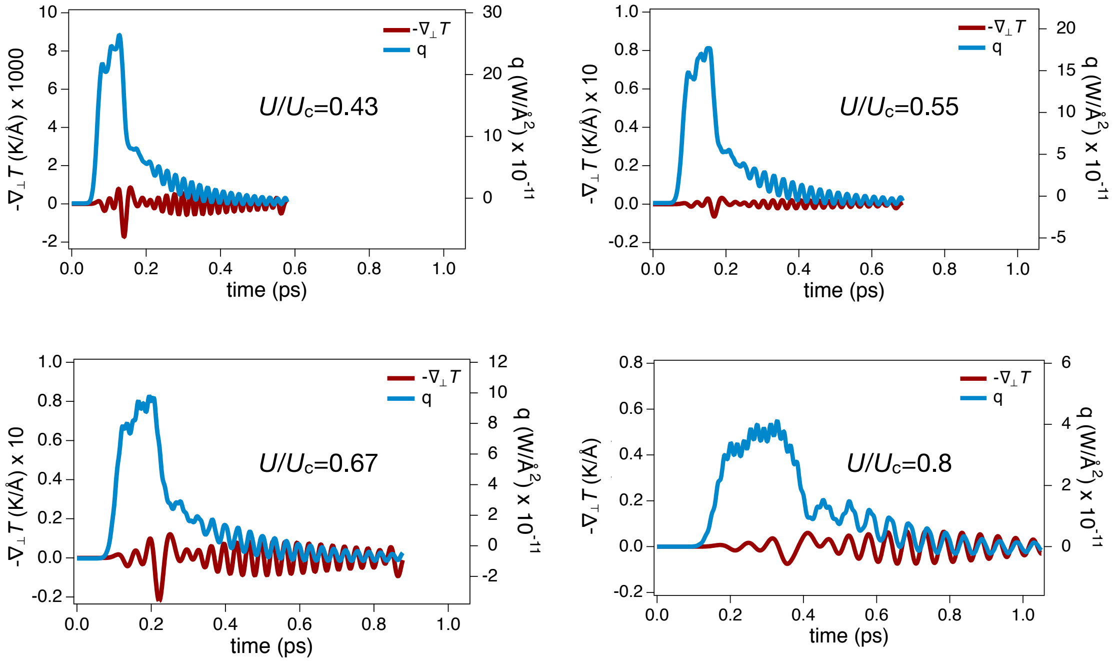

Supplementary Figure 3: Dynamics of the interlayer temperature gradient -$\nabla_\perp T(t)$ (purple line) and heat flux $q(t)$ (blue line) at the 15th layer for different values of U/U_c. The data reported in the figure have been used to retrieve the electronic thermal conductivity in the Fourier-like regime.

SUPPLEMENTARY REFERENCES

[1] M. Gandolfi, G. Benetti, C. Glorieux, C. Giannetti, and F. Banfi, International Journal of Heat and Mass Transfer **143**, 118553 (2019).
[2] L. Zhang, Y. Zhou, L. Guo, W. Zhao, A. Barnes, H.-T. Zhang, C. Eaton, Y. Zheng, M. Brahlek, H. F. Haneef, N. J. Podraza, M. H. W. Chan, V. Gopalan, K. M. Rabe, and R. Engel-Herbert, Nature Materials **15**, 204 (2015).
[3] S. Aizaki, T. Yoshida, K. Yoshimatsu, M. Takizawa, M. Minohara, S. Ideta, A. Fujimori, K. Gupta, P. Mahadevan, K. Horiba, H. Kumigashira, and M. Oshima, Phys. Rev. Lett. **109**, 056401 (2012).
